# Levansucrase from *Bacillus amyloliquefaciens* KK9 and Its Y237S Variant Producing the High Bioactive Levan-Type Fructooligosaccharides

**DOI:** 10.3390/biom10050692

**Published:** 2020-04-29

**Authors:** Pongsakorn Phengnoi, Thanapon Charoenwongpaiboon, Karan Wangpaiboon, Methus Klaewkla, Santhana Nakapong, Wonnop Visessanguan, Kazuo Ito, Rath Pichyangkura, Kamontip Kuttiyawong

**Affiliations:** 1 Department of Chemistry, Faculty of Liberal Arts and Science, Kasetsart University, Kamphaeng Saen Campus, Nakhon Pathom 73140, Thailand; pkphe@hotmail.com; 2 Department of Chemistry, Faculty of Science, Silpakorn University, Nakhon Pathom 73000, Thailand; thanapon.charoenwongpaiboon@gmail.com; 3 Department of Biochemistry, Faculty of Science, Chulalongkorn University, Bangkok 10330, Thailand; wangpaiboon9@gmail.com (K.W.); methus.kanon@gmail.com (M.K.); prath@chula.ac.th (R.P.); 4 Department of Chemistry, Faculty of Science, Ramkhamhaeng University, Bangkok 10240, Thailand; santhana@ru.ac.th; 5 National Center for Genetic Engineering and Biotechnology, National Science and Technology Development Agency, Pathumthani 12120, Thailand; wonnop@biotec.or.th; 6 Graduate School of Science, Osaka City University, Osaka 558-8585, Japan; ito@sci.osaka-cu.ac.jp

**Keywords:** *Bacillus amyloliquefaciens*, fructooligosaccharide, levansucrase, prebiotic, mutagenesis

## Abstract

Levan-typed fructooligosaccharide (LFOS), a β-2,6 linked oligofructose, displays the potential application as a prebiotic and therapeutic dietary supplement. In the present study, LFOS was synthesized using levansucrase from *Bacillus amyloliquefaciens* KK9 (LsKK9). The wild-type LsKK9 was cloned and expressed in *E. coli*, and purified by cation exchanger chromatography. Additionally, Y237S variant of LsKK9 was constructed based on sequence alignment and structural analysis to enhance the LFOS production. High-performance anion-exchange chromatography coupled with pulsed amperometric detection (HPAEC-PAD) analysis indicated that Y237S variant efficiently produced a higher amount of short-chain LFOS than wild type. Also, the concentration of enzyme and sucrose in the reactions was optimized. Finally, prebiotic activity assay demonstrated that LFOS produced by Y237S variant had higher prebiotic activity than that of the wild-type enzyme, making the variant enzyme attractive for food biotechnology.

## 1. Introduction

Fructooligosaccharide (FOS) is a well-known prebiotic which is widely used in food and pharmaceutical industries. Besides prebiotic, FOS was also used as a sweetener and dietary supplement for low-carbohydrate consumers [1,2]. FOS can be divided into 2 major categories based on the linkages between the fructosyl unit. Inulin-typed fructooligosaccharide (IFOS) is mainly linked by β-2,1linkage, while major linkage in levan-typed fructooligosaccharide (LFOS) consist of β-2,6 bound.

**Figure 1:**
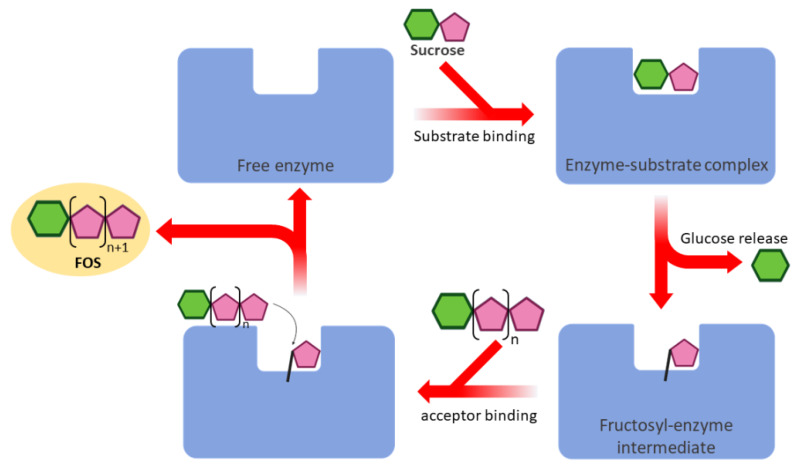
Schematic illustration of the reactions occurring in the active site of fructosyltransferase.

Typically, FOS was synthesized by enzymatic reactions. Hydrolysis is the reactions used to produce FOS from fructan polysaccharides. This reaction can be achieved by inulinase (EC 3.2.1.7) and levanase (EC 3.2.1.65), the enzymes randomly hydrolyzed β-2,1 and β-2,6 glycosidic linkages of fructan, respectively [3,4]. However, the production of FOS by hydrolysis was limited because it is difficult to control the molecular weight of FOS produced. On the other hand, FOS can be synthesized from the small molecule of saccharides by transglycosylation reaction. Fructosyltransferases are enzymes that can transfer the fructosyl units from “glycosyl donor” to “acceptor”. In principle, the synthesis of FOS is divided into two steps: initially, sucrose molecules (glycosyl donor) are split, and the fructosyl-enzyme intermediate is formed; then, the acceptor molecules attach to the fructosyl-enzyme intermediate, and FOS are formed (Figure 1). There are two kinds of fructosyltransferases; inulosucrase (EC 2.4.1.9) and levansucrase (EC 2.4.1.10), which produced inulin and levan from sucrose, respectively. Although inulosucrase and levansucrase shared very high amino acid sequence and structure similarity, these enzymes exhibited the different mechanism for product elongation and linkage formation [5,6,7].

Levansucrase was found in many bacteria species, such as *Bacillus subtilis* (Bs_SacB) [8], *Bacillus megaterium* (Bm_SacB) [9], *Bacillus licheniformis* (Bl_SacB) [10] and *Lactobacillus reuteri* [11]. Both free and immobilized levansucrase have been used to produce the LFOS [10,11,12]. However, the production of LFOS by levansucrase is not effective since the main product is polysaccharides. Many studies attempted to modulate the size distribution of levan using site-directed mutagenesis. According to Strube et al. [9], the degree of polymerization of LFOS produced by *B. megaterium* levansucrase can be changed by mutation at residues on the substrate-binding cavity. Since the prebiotic effect of FOS strongly relied on a degree of polymerization (DP) [2] and the LFOS displays superior prebiotic activity and chemical stability compared to IFOSs [13,14,15], variant levansucrases producing short-chain levan should be useful for dietary supplement production.

We herein describe the cloning, expression and site-directed mutagenesis of levansucrase from *Bacillus amyloliquefaciens* KK9 (LsKK9). Y237S variant LsKK9 was constructed in order to improve the yield of LFOS produced. The influences of reaction conditions, including sucrose and enzyme concentration, on LFOS synthesis, were also investigated and compared to the wild-type enzyme. Finally, the prebiotic activity of LFOS produced from Y237S variant was determined and compared to that of wild type.

## 2. Materials and Methods

### 2.1. Cloning of Levansucrase from Bacillus Amyloliquefaciens KK9

The *B. amyloliquefaciens* KK9, isolated from soil in Thailand and identified by 16S rRNA sequence, was grown aerobically in LB broth at 37 °C. The gene encoding levansucrase was amplified from the chromosomal DNA of *B. amyloliquefaciens* KK9 using the partial degeneracy primers which were designed based on sequence alignment; forward primer (5′-CATGCCATGGACATCAAAAAG(A)T(A)TTGC(T)AAAAC-3′) and reverse primer (5′-CGGGATCCTTATTG(A,T)GTTA(G)ACTGTT(C)AA(G)T(C)TGTCCTTG-3′). Pfu polymerase (Promega™) was used for DNA amplification. The PCR product was ligated into pET-19b vector (Novagen™) via NcoI and BamHI sites and transformed into *E. coli* Top10 (Invitrogen™). The sequence of recombinant plasmid was verified by nucleotide sequencing and assigned to GenBank in Accession number KC477262.

### 2.2. Expression and Purification of Recombinant Levansucrase

The sequence-verified plasmid was transformed into *E. coli* BL21 Star^TM^ (DE3) (Invitrogen™, Waltham, MA, USA). The recombinant cells were cultured in Luria-Bertani (LB) medium containing 0.1 mg/mL ampicillin and 1% (*w/v*) glucose at 37 °C, 250 rpm until OD600 reach 0.4-0.6. After that, IPTG was added to the culture medium to a final concentration of 0.1 mM, and further incubated at 30 °C, 250 rpm for overnight. The cells were harvested by centrifugation at 8000× *g*, 4 °C for 10 min, and resuspended in lysis buffer (50 mM acetate buffer (pH 6.0) containing 0.1%TritonX-100, and then lysed by ultrasonication (35% amplitude for 2 min with pulse durations of 1 s on and 2 s off, Sonics Vibra-Cell™). The cell debris was discarded by centrifugation at 10,000× *g* for 15 min.

A crude extract of a recombinant levansucrase (LsKK9) was loaded onto hand-packed TOYOPEARL CM-650 column (column volume 20 mL; Tosoh Bioscience™, Tokyo, Japan) pre-equilibrated with 200 mL of acetate buffer (50 mM; pH 6.0) at 0.7 mL/min and 4 °C. The column was washed by the same buffer (~10 column volume) until the OD_280_ reach baseline and then eluted by a stepwise gradient of acetate buffer (50 mM; pH 6.0) containing 100 mM, 150 mM and 300 mM NaCl, respectively. The fractions with levansucrase activity were collected and pooled. The purity of enzymes was evaluated by SDS-PAGE and purification table (Table S1).

### 2.3. Enzyme Activity Assay

The activity of both wild-type and variant LsKK9 was determined by 3,5-dinitrosalicylic acid method [16]. The enzyme was incubated with 5% (*w/v*) sucrose in acetate buffer pH 6.0 at 37 °C in 0.5 mL total volume. Subsequently, the reactions were terminated by adding 0.5 mL DNS reagent and then boiled for 10 min. The amount of reducing sugar was determined by spectrophotometer at A_540_. Standard glucose was used as a calibration curve. One unit of the enzyme was defined as the amount of levansucrase that released one µmole of reducing sugar per min.

### 2.4. Amino Acid Sequence Alignment and Homology Modelling Study

Multiple amino acid sequence alinement of wild-type LsKK9 was conducted using Clustal Omega [17]. The docking study was performed using homology models of both wild-type and Y237S variant LsKK9 and pentasaccharide of LFOS (GF4). Glycosciences.DB server [18] was employed to construct the structure of GF4 with a sequence of [β-d-Fruf-(2-6)-β-d-Fruf-(2-6)-β-d-Fruf-(2-1)-β-d-Fruf-(2-1)-α-D-Glcp], the main pentasaccharide product of levansucrase based on a previous study [19]. Homology models of both wild-type and Y237S variant LsKK9 were constructed using SWISS-MODEL server [20] using a crystal structure of *B. subtilis* levansucrase variant E342A bound to raffinose (PDB ID: 3BYN) [21] as a template (89% identity and 98% coverage). Ramachandran plots were then produced using the same server to validate the quality of the homology models [22]. Most residues are mainly present in a favored region with 95.43% (Figure S1). Binding conformation of GF4 in the binding site of both wild-type and Y237S variant LsKK9 was predicted using Autodock Vina [23]. To construct the enzyme-GF4 complex, GF4 was docked into the binding site of both wild-type and Y237S variant LsKK9 models using a grid box of 30 Å × 30 Å × 30 Å with a grid spacing of 1Å.

### 2.5. Product Characterization

#### 2.5.1. NMR Spectroscopy

The polysaccharide product was harvested from the reaction by ethanol precipitation and further purified by BioGel P-100 (Bio-Rad, Hercules, CA, USA) using DI water as eluent. The levan was dissolved in D_2_O, and the spectra were acquired at 40 °C on a Varian VNMRS-500 operating at 125 MHz for ^13^C and 500 MHz for ^1^H. Chemical shifts were measured relative to dimethylsilapentane-5-sulphonate (DSS) as the internal standard.

#### 2.5.2. Thin-layer Chromatography (TLC)

The TLC analysis was performed according to the method described previously [24]. The mobile phase consisted of 1-butanol: glacial acetic acid: water, 3:3:2 (*v/v/v*). The separation was conducted for 3 ascents using TLC silica gel 60 F254 (Merck™). The TLC plates were dried and stained with a solution containing 8 mL of water, 10 mL of concentrated H_2_SO_4_, 27 mL of ethanol and 0.1 g of orcinol. The TLC were visualized by heating at 110 °C for 10 min.

#### 2.5.3. High-performance Liquid Chromatography (HPLC)

The transglycosylated product (fructan + LFOS) was analyzed by HPLC equipped with a refractive index detector (Shimadzu™ Corporation, Kyoto, Japan). The product was separated by Sugarpak column (Water™, Milford, MA, USA) using 50 mg/L CaEDTA as mobile phase, at a flow rate of 0.5 mL/min and 70 °C. Glucose, fructose and sucrose were used as external standards for mono- and di-saccharide quantification. The amount of transglycosylated product was calculated using following equation: Total FOS (% (*w/v*)) = initial sucrose (% (*w/v*)) − remaining sucrose (% (*w/v*)) − glucose (% (*w/v*)) − fructose (% (*w/v*)).

#### 2.5.4. High-performance Anion-Exchange Chromatography Coupled With Pulsed Amperometric Detection (HPAEC-PAD)

The pattern of oligosaccharides produced by the enzymes was analyzed by Dionex™ ICS 5000 system using CarboPac™ PA-1 column (Thermo™). Initially, the column was equilibrated with 150 mM NaOH at the flow rate of 1 mL/min. Then, the samples were injected and eluted by a linear gradient of 0 to 300 mM sodium acetate in 150 mM NaOH for 25 min. After that, the concentration of sodium acetate was sharply increased from 300 to 500 mM within a minute and held for 4 min before going down to equilibrated condition.

### 2.6. Site-directed Mutagenesis

Site-directed mutagenesis was achieved by PCR-driven overlap technique [25]. The primers F_Y237S (5′-CGATGAAGGCAACAGCACATCCGGCGACAACC-3′) and R_Y237S (5′-GGTTGTCGCCGGATGTGCTGTTGCCTTCATCG-3′) were designed for mismatch mutation and was amplified by PrimeStar™ polymerase (Takara Bio^TM^, Shiga, Japan). The PCR product was recombined into pET-19b expression vector via NcoI and BamHI. The point mutation was verified by DNA sequencing.

### 2.7. Biochemical Characterization

The effect of pH and temperature on the initial rate of both wild-type and Y237S variant LsKK9 was measured using DNS assay. For the effect pH on enzyme activity, relative enzymatic activity was measured at 37 °C in 20 mM Biston-Robinson’s universal buffer pH range of 3.0–8.0, while the effect of temperature was monitored in 50 mM citrate buffer pH 6.0 at the temperature of 4 to 60 °C. All experiments were performed in triplicate.

### 2.8. Synthesis of LFOS Using Wild-Type and Variant LsKK9

LFOS was synthesized from sucrose using both wild-type and Y237S variant LsKK9. The reactions were conducted at 37 °C for 24 h. The effect of enzyme concentration on LFOS synthesis was investigated using 0.5–3 U/mL of enzymes, while sucrose concentration was constant at 10% (*w/v*). Likewise, the effect of sucrose concentration on LFOS synthesis was explored by incubating 2 U/mL of enzymes with 5–50% (*w/v*) of sucrose. After incubation, the reactions were terminated by boiling for 10 min, and their carbohydrate compositions were analyzed by HPLC as described above.

After that, the obtained LFOS was purified by Bio-Gel P2 column (Bio-Rad™) using ultrapure water as mobile phase. LFOS was separated by this column at 50 °C using a flow rate of 0.6 mL/min. The fractions containing LFOS were pooled and lyophilized.

### 2.9. Prebiotic Activity Study

*Lactobacillus plantarum* NCIMB 8826 and *Escherichia coli* ATCC 35401 was used for prebiotic activity study. The cells were statically cultured in 5 mL of MRS broth medium at 37 °C for 48 h. After that, the cells were harvested by centrifugation at 7500× *g* for 10 min, and washed by 0.85% (*w/v*) NaCl for 2 times. Then the cells were cultured in 2.5 mL MRS broth containing 2% (*w/v*) of different carbon sources, namely glucose, wild-type LFOS and Y237S LFOS, using final cell concentration of 5-6 Log CFU/mL. After cultivation at 37 °C for 48 h, the diluted cells were spread on MRS agar medium (for *L. plantarum*) or LB agar medium (for *E. coli*), and incubated at 37 °C for 48 h. The growth of bacteria was explored by counting the number of colonies forming after incubation. Prebiotic activity score of each carbohydrate sources can be calculated using the following equation [26,27].

Prebiotic activity score = [(PP_48h_ − PP_0h_)/(PG_48h_ − PG_0h_)] − [(EP_48h_ − EP_0h_)/(EG_48h_ − EG_0h_)] (1)

were PP_0h_, PP_48h_, PG_0h_ and PG_48h_ are the number of colonies of *L. plantarum* (Log CFU/mL) cultured in MRS containing target prebiotic samples (PP) or glucose (PG) at 0 and 48 h, whereas EP_0h_, EP_48h_, EG_0h_ and EG_48h_ are the number of colonies of *E.coli* (Log CFU/mL) cultured in MRS containing target prebiotic samples (EP) or glucose (EG) at 0 and 48 h.

## 3. Results and Discussion

### 3.1. Cloning, Expression and Product Characterization of Levansucrase from Bacillus Amyloliquefaciens KK9

**Figure 2:**
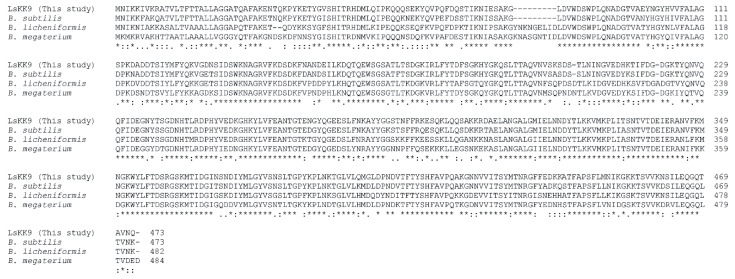
Sequence alignment of wild-type LsKK9 and other levansucrases. Sequences were compared with the following enzyme, source and accession numbers: Bs_SacB, *B. subtilis* subsp. *subtilis* str. 168 (GenBank: CAA26513.1); Bl_SacB, *B. licheniformis* 8-37-0-1 (GenBank: AGZ16261); Bm_SacB, *B. megaterium* DSM 319 (GenBank: ADF38395.1). The alignment was performed using Clustal Omega.

Levansucrase from *Bacillus amyloliquefaciens* KK9 (LsKK9) was successfully cloned and showed 1422 bp of ORF encoding 473 amino acid residues, with theoretical molecular mass 52,974.12 Da. Although levansucrase from *B. amyloliquefaciens* has been reported from other isolations [28,29], the wild-type LsKK9 has a minor variation of amino acid sequence approximately 2%. Additionally, the sequence alignment showed that LsKK9 sequence showed 90%, 76%, and 72% sequence identity with those of Bs_SacB, Bl_SacB, and Bm_SacB, respectively (Figure 2). The wild-type LsKK9 was overexpressed in *E. coli* BL21 Star^TM^ (DE3) with a specific activity of 82 U/mg protein (Table S1). After purification by cation chromatography, the specific activity increased to 152 U/mg protein. The recovery of recombinant wild-type LsKK9 is 55.2% of the total activity with 1.9-fold purification. Approximately 90% purity of the enzyme was obtained as shown in SDS PAGE (Figure S2).

**Figure 3:**
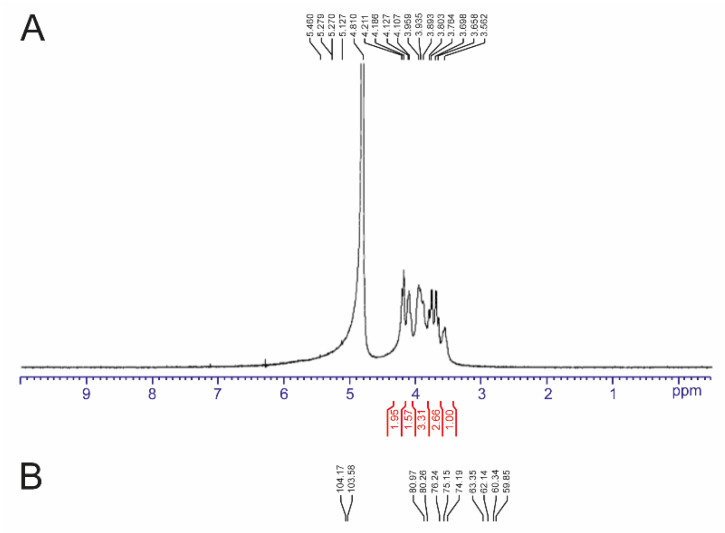
(**A**) ^1^H-NMR and (**B**) ^13^C-NMR spectrum of the polymer synthesized by wild-type LsKK9.

Structure of the obtained polysaccharide was analyzed by ^13^C-NMR and ^1^H-NMR. As shown in Figure 3, the ^1^H-NMR spectrum showed seven protons between 3.8 and 4.8 ppm (Figure 3A). For ^13^C-NMR spectrum, six main resonances at 104.2 (C2), 80.3 (C5), 76.2 (C3), 75.1 (C4), 62.1 (C6) and 59.9 (C1) ppm were observed (Figure 3B). These chemical shifts were characteristic of β-configurated fructosyl units, which are almost identical to the levan product of other bacteria sources (Table 1). This data confirmed the product as a levan type-fructan.

### 3.2. Molecular Docking Study

**Figure 4:**
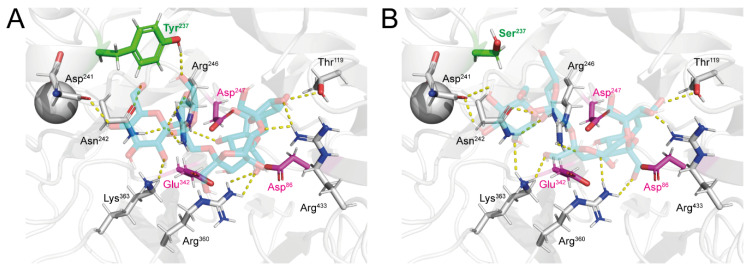
The docking conformation of GF4 into the (**A**) wild-type and (**B**) Y237S mutant LsKK9. The catalytic triad of the enzyme was shown in magenta, and the position of the mutated amino acid residue (Tyr^237^ and Ser^237^) was shown in green.

Based on the sequence alignment and 3D structure of homology modelling, the catalytic residues of LsKK9 were proposed to be Asp^86^ (nucleophile), Asp^247^ (transition-state stabilizer), and Glu^342^ (general acid-base catalyst) (Figure 4). According to Strube et al. (2011) [9], mutagenesis at Tyr^247^, Asn^252^, and Lys^373^ of Bm_SacB affected the polysaccharide formation activity and oligosaccharide spectrum. Therefore, Ty^r237^, Asn^242^, and Lys^363^ (equivalent to Tyr^247^, Asn^252^, and Lys^373^ of Bm_SacB [9]) should be a substrate-entering channel of wild-type LsKK9, which remotely located from the catalytic triad. The molecular docking study was also performed in order to investigate the binding conformation of the substrate in the pocket of both wild-type and Y237S variant LsKK9. The result suggests that GF4 positioned on the predicted substrate-binding pocket previously described by Strube et al. [9], and formed hydrogen bonds with many amino acid residues, including Tyr^237^, Asn^242^ and Lys^363^ (equivalent to Tyr^247^, Asn^252^, and Lys^373^ of Bm_SacB from Strube et al. (2011) [9]) (Figure 4A). On contrary, the Y237S variant LsKK9 lose their hydrogen bond between Tyr^237^ and GF4 after this residue was replaced by Serine (Figure 4B). This is along with the reduction of free binding energy for GF4 from -7.7 kcal/mol in the wild type to -7.1 kcal/mol in the variant. This result suggested that the Y237S variant might synthesize the short chain fructan.

### 3.3. Site-Directed Mutagenesis and Biochemical Characterization

According to our previous study, mutation at Tyr^246^ of *B. licheniformis* RN-01 levansucrase (equivalent to Tyr^237^ in LsKK9) strongly affected the enzyme activity and the degree of polymerization (DP) of levan product [12,34]. Nevertheless, Y246S displayed a potential application to produce a high amount of LFOS with slight levan polymer. Moreover, this mutant synthesized the LFOS with DP3 – 10, which potentially be used as a prebiotic [35,36]. Therefore, in this study, Y237S variant of LsKK9 was constructed to improve the yield of prebiotic LFOS.

**Figure 5:**
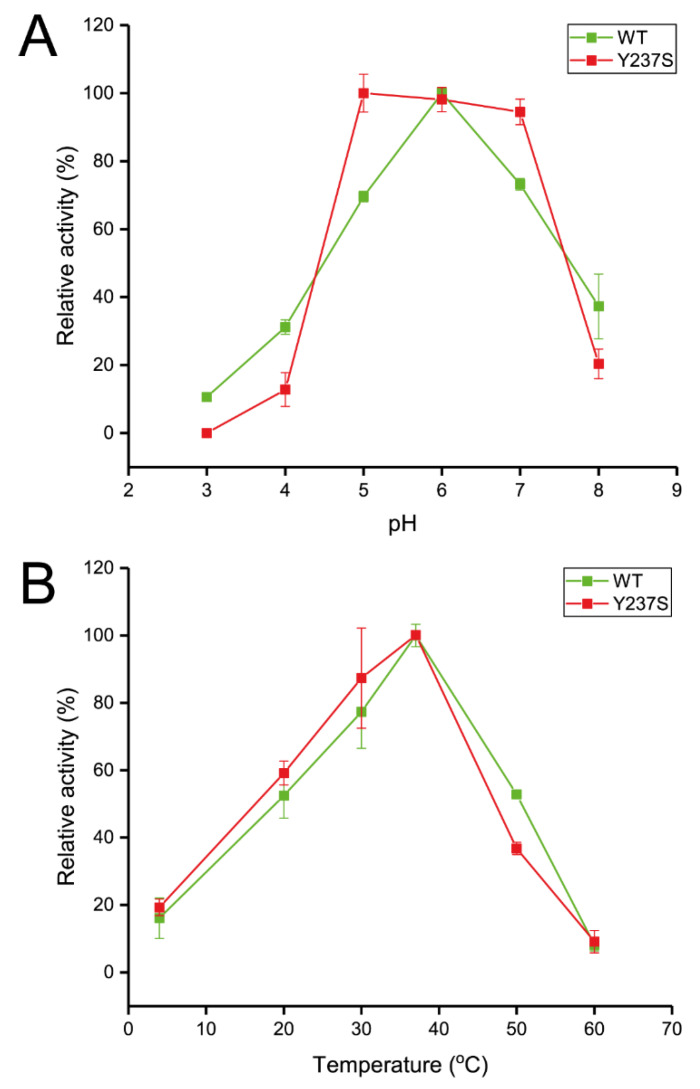
Effect of pH (**A**) and temperature (**B**) of wild-type and Y237S variant LsKK9.

Afterwards, variant enzymes were expressed and purified in the same condition with the wild type. Subsequently, activity and biochemical properties of the variant enzymes were analyzed and compared. The specific activity of Y237S varaint was 104 U/mg protein (Table S1), which decreased around 1.5-fold compared with that of the wild type (152 U/mg protein). The reduction of activity could usually be observed after mutation at acceptor binding residues as confirmed by several reports [5,10]. The effect of pH and temperature on the activity of wild-type and the variant enzymes were also investigated. Surprisingly, the Y237S variant showed broad optimum pH ranging from pH 5.0–7.0, while wild type showed a narrow curve and peaked at pH 6.0 (Figure 5A). However, mutation at Tyr^237^ did not significantly affect the optimum temperature for wild-type LsKK9 activity (37 °C) (Figure 5B). The optimum pH and temperature for wild-type LsKK9 are in acceptable range with several levansucrases which usually displayed activity optima at neutral range (pH 6.0–7.0), and temperatures optima between 30 and 45 °C.

### 3.4. Production of LFOS by Wild-Type and Y237S Variant LsKK9

**Figure 6:**
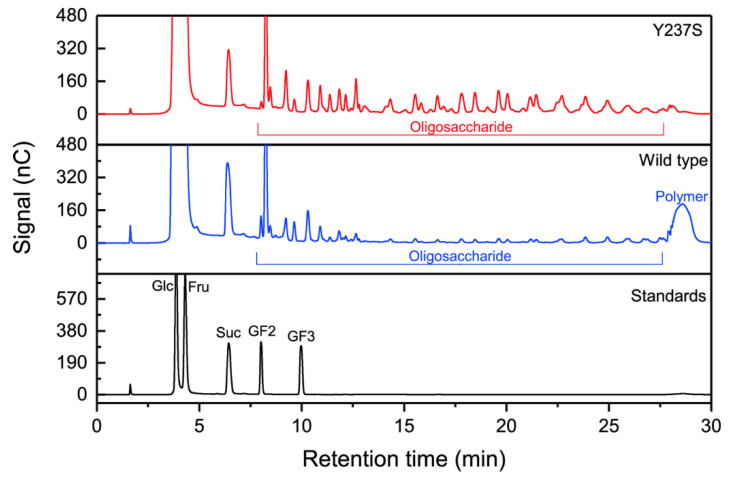
HPAEC chromatogram of wild-type and Y237S variant LsKK9.

Both wild-type and Y237S were used for LFOS production using sucrose substrate. The pattern of LFOS was explored using HPAEC-PAD. As shown in the chromatogram (Figure 6), Y237S product profiles relatively exhibited a high amount of oligosaccharides than that of wild type, while the peak of polysaccharide at 29 min of retention time cannot be observed. In addition, most peaks from Y237S products were also present similar to in that of wild-type LsKK9. This indicated that the mutation at Tyr237 did not impact on overall linkages of obtained oligosaccharides. As a result, the substitution of Tyr^237^ by Ser could enhance the yield of oligosaccharides and suppressed polysaccharide forming of levansucrase, which is correlated to previous studies [12,34,37].

**Figure 7:**
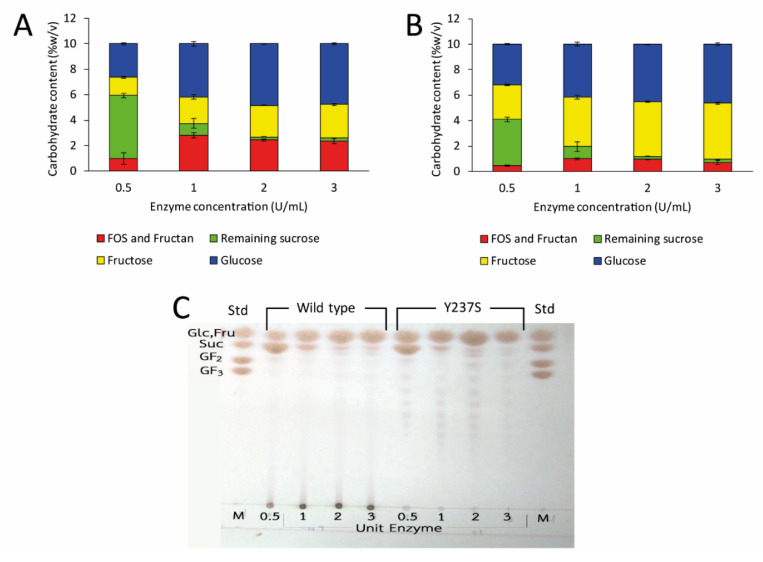
Effect of enzyme concentration on LFOS product. The amount of fructose, glucose, remaining sucrose and transglycosylated products produced by (**A**) wild-type and (**B**) Y237S variant LsKK9 was analyzed by HPLC. (**C**) LFOS profiles were analyzed by TLC.

To achieve the highest yield of LFOS, the reaction conditions were subsequently optimized. In this study, the effect of enzyme and sucrose concentration on the yield of LFOS was explored, whereas pH and temperature of the reaction were fixed at pH 6.0 and 37 °C. For the effect of enzyme concentration, the units of the enzyme were varied from 0.5 to 3 units per mL of reaction. As a result, the LFOS synthesis was largely dependent on the enzyme concentration. The concentration of transglycosylated products (Fructan + LFOS) increased when enzyme concentration increased and reached to the highest yield at 1 U/mL (Figure 7A,B). However, when considering in term of the percent sucrose conversion, >95% conversion sucrose was completely transformed when at least 2 U/mL of enzymes were used. Thus, two units per mL condition were selected for further analysis since it provided the low concentration of sucrose, preventing the contamination of LFOS by the high amount of disaccharide. TLC analysis showed that Y237S variant mainly produced LFOS, while wild type mainly synthesized polysaccharide (Figure 7C).

**Figure 8:**
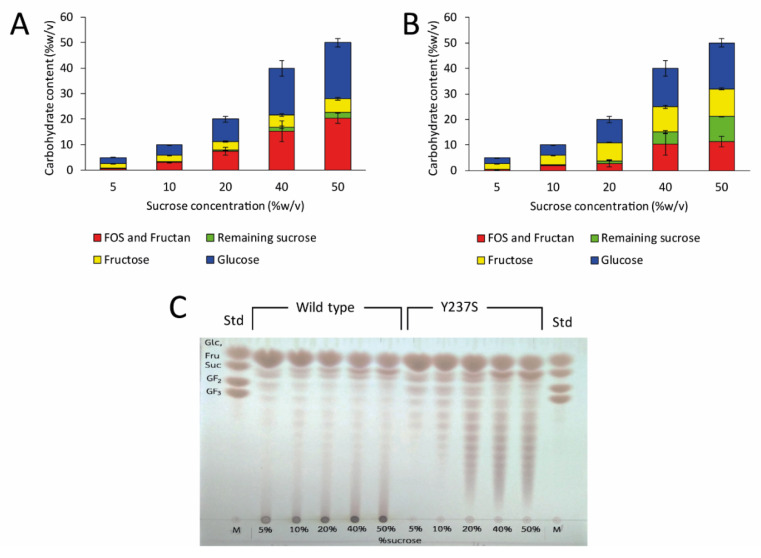
Effect of sucrose concentration on LFOS product. The amount of fructose, glucose, remaining sucrose and transglycosylated products produced by (**A**) wild-type and (**B**) Y237S variant LsKK9 was analyzed by HPLC. (**C**) LFOS profiles were analyzed by TLC.

Then, the effect of sucrose concentration on LFOS production was explored, using sucrose at final concentrations of 5–50% (*w/v*). The results showed that the amount of transglycosylated product increased when sucrose concentration was risen (Figure 8A,B). The concentration of transglycosylated products produced by wild-type LsKK9 increased up to 20% (*w/v*) when 50% (*w/v*) sucrose was used. In the case of Y237S, the concentration of transglycosylated products increased and reached a plateau when ≥ 40% (*w/v*) sucrose was used. Although wild-type enzyme produced higher transglycosylated products, the main product is a polysaccharide. On the other hand, Y237S produced mainly LFOS with a slight polymer (Figure 8C). As a result, we can conclude that Y237S variant LsKK9 has a potential for production of LFOS, which might be used as a prebiotic.

### 3.5. Prebiotic Activity

**Figure 9:**
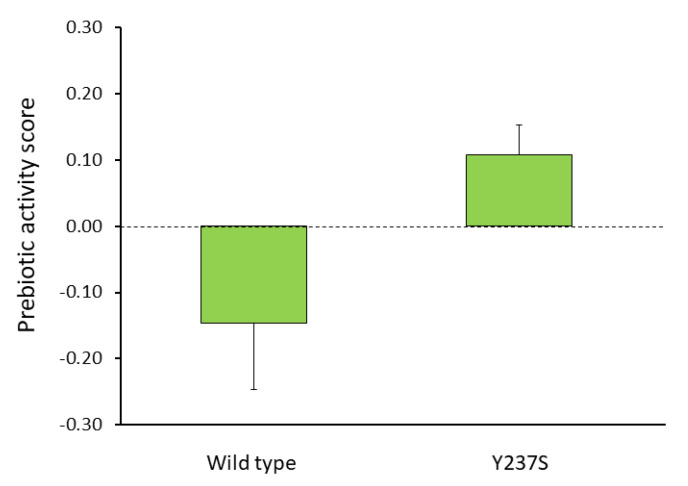
The prebiotic activity of levan and LFOS synthesized by wild-type and variant LsKK9.

Although the mutation at equivalent Y237 position of other levansucrases have previously been reported [9,34,37], the biological properties of their products have never been evaluated compared with that of wild-type enzyme. Therefore, LFOS synthesized by both wild-type and Y237S variant LsKK9 were purified by Bio-gel P2 column size exclusion chromatography to eliminate mono- and disaccharide in the reaction mixture. Subsequently, the prebiotic activity of the purified LFOSs was evaluated using *Escherichia coli* ATCC 35401 and *Lactobacillus plantarum* NCIMB 8826. The prebiotic activity score of wild-type LFOS is approximate of -0.15, whereas that of Y237S is 0.11 (Figure 9). It indicated that LFOS produced by Y237S variant had higher prebiotic activity than products from wild type. This finding corresponded to the previous study that lower DP fructans strongly stimulated the growth of *Lactobacilli* and other probiotics [35,36].

## 4. Conclusions

This study illustrated the rational mutagenesis of levansucrase from *Bacillus amyloliquefaciens* KK9 to increase the yield of short-chain LFOS and suppress polysaccharide production. The substitution at Y237 by serine did not only affect biochemical properties of LsKK9, but also increase the prebiotic activity of LFOS produced. These properties make the Y237S variant LsKK9 highly attractive as an alternative catalyst for the production of the prebiotic and for biotechnological applications.

## Figures and Tables

**Table 1:** Comparison of the ^13^C-NMR chemical shifts of the levan produced from different bacterial sources.

Sources	Chemical Shifts (ppm)						References
	C-1	C-2	C-3	C-4	C-5	C-6	
*B. polymyxa*	60.7	104.2	77	75.7	80.5	63.6	[30]
*B. subtilis*	60.1	104.4	76.5	75.4	80.5	63.6	[31]
*B. licheniformis*	62.9	106.9	79.3	78.1	83	66.1	[32]
*B. aryabhattai*	62.7	106.9	79	77.9	83	66.1	[33]
*B. amyloliquefaciens KK9*	59.9	104.2	76.2	75.1	80.3	62.1	This study

## Supplementary Materials

The following are available online at https://www.mdpi.com/2218-273X/10/5/692/s1, Figure S1: Ramachandran Plot for homology modelling of LsKK9, Figure S2: SDS-PAGE of purified wild-type and Y237S mutant LsKK9, Table S1: LsKK9 purification data.
