# Supplementary material for: Levansucrase from Bacillus amyloliquefaciens KK9 and Its Y237S Variant Producing the High Bioactive Levan-Type Fructooligosaccharides
Source: Biomolecules. 2020 Apr 29;10(5):692. doi: 10.3390/biom10050692 (PMC7277640; doi:10.3390/biom10050692)
Supplement: Supplementary file 1 [file biomolecules-10-00692-s001.pdf]

Supporting information

Levansucrase from *Bacillus amyloliquefaciens* KK9 and its Y237S variant producing the high bioactive levan-type fructooligosaccharides

Pongsakorn Phengnoi [1], Thanapon Charoenwongpaiboon [2], Karan Wangpaiboon [3], Methus Klaewkla [3], Santhana Nakapong [4], Wonnop Visessanguan [5], Kazuo Ito [6], Rath Pichyangkura [3] and Kamontip Kuttiyawong [1,*]

[1] Department of Chemistry, Faculty of Liberal Arts and Science, Kasetsart University, Kamphaeng Saen Campus, Nakhon Pathom, 73140, Thailand; pkphe@hotmail.com

[2] Department of Chemistry, Faculty of Science, Silpakorn University, Nakhon Pathom, 73000, Thailand; thanapon.charoenwongpaiboon@gmail.com

[3] Department of Biochemistry, Faculty of Science, Chulalongkorn University, Bangkok, 10330, Thailand; wangpaiboon9@gmail.com (K.W.); methus.kanon@gmail.com (M.K.); prath@chula.ac.th (R.P.)

[4] Department of Chemistry, Faculty of Science, Ramkhamhaeng University, Bangkok, 10240, Thailand; santhana@ru.ac.th

[5] National Center for Genetic Engineering and Biotechnology, National Science and Technology Development Agency, Pathumthani, 12120, Thailand; wonnop@biotec.or.th

[6] Graduate School of Science, Osaka City University, Osaka, 558-8585, Japan; ito@sci.osaka-cu.ac.jp

* Correspondence: kamontip.k@ku.ac.th.

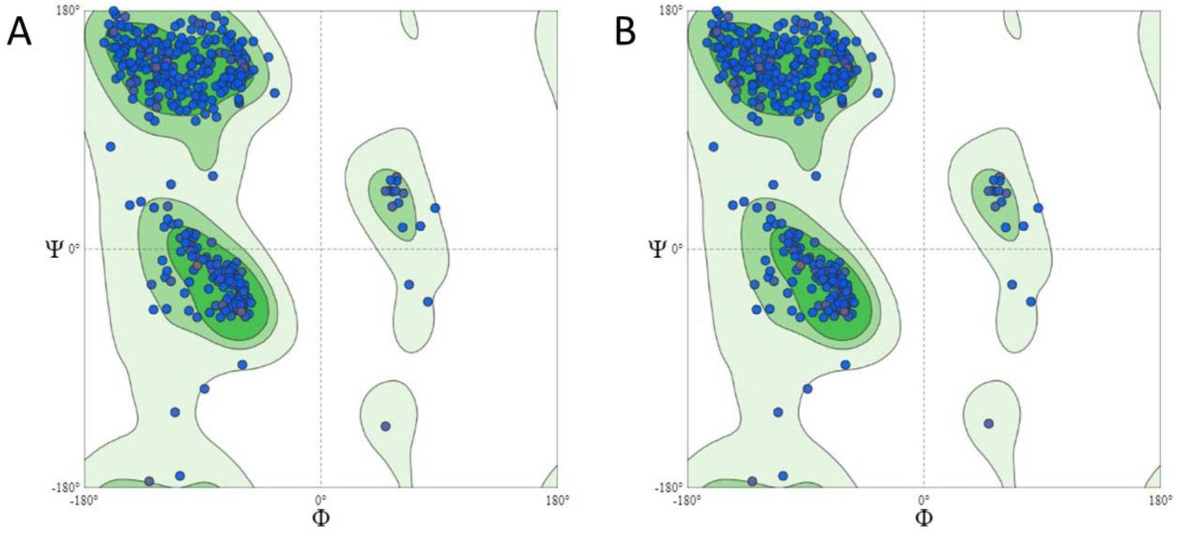

Figure S1. Ramachandran Plot for homology modeling of (A) wild-type and (B) Y237S mutant LsKK9.

Table S1. LsKK9 purification data.

Step	Total activity (U)	Total protein (mg)	Specific activity (U/mg)	Fold	Recovery (%)
Wild type					
Crude	1850	22.6	81.9	-	100
CM toyopearl	1021	6.7	152.4	1.9	55
Y237S					
Crude	2368	25.9	91.4	-	100
CM toyopearl	969	9.4	103.5	1.1	41

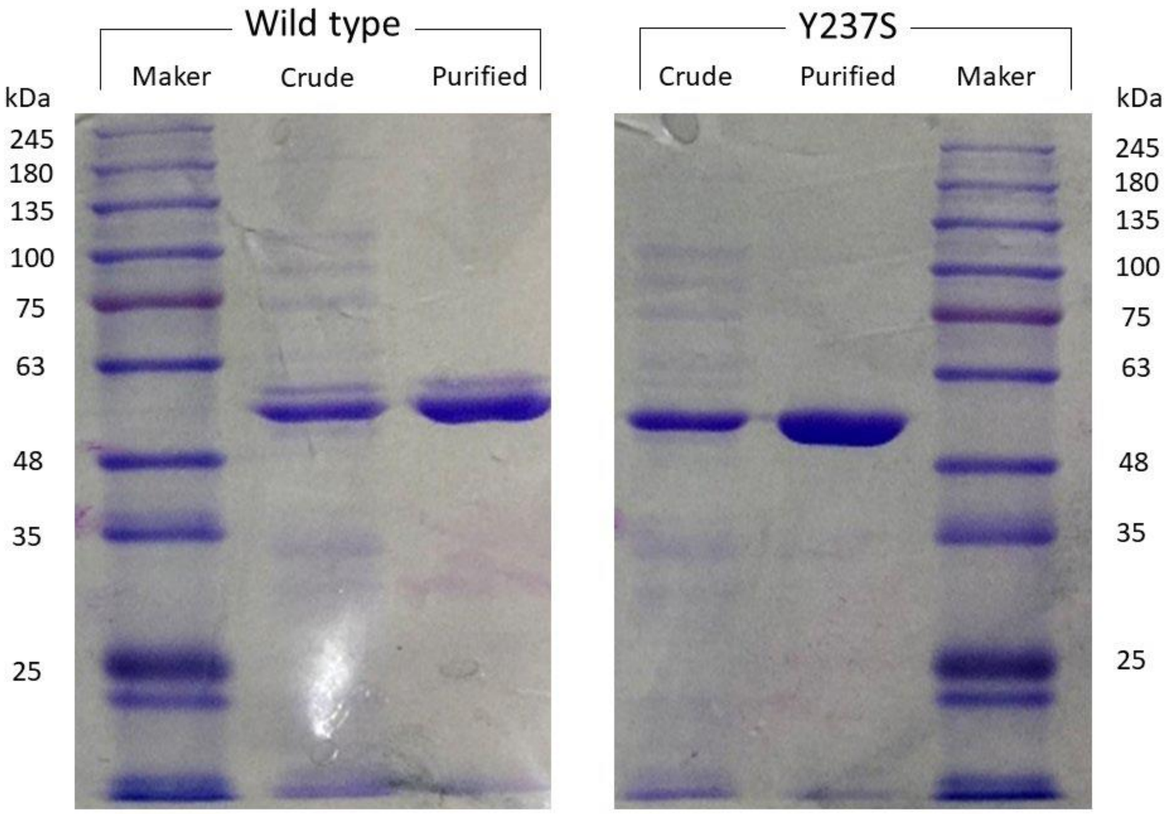

Figure S2. SDS-PAGE of purified wild-type and Y237S mutant LsKK9.
